# The Relationship Between Sleep Quality and Internet Addiction Among Female College Students

**DOI:** 10.3389/fnins.2019.00599

**Published:** 2019-06-12

**Authors:** Pin-Hsuan Lin, Ya-Chen Lee, Kai-Li Chen, Pei-Lun Hsieh, Shang-Yu Yang, Ying-Lien Lin

**Affiliations:** ^1^Department of Health and Beauty, Shu-Zen Junior College of Medicine and Management, Kaohsiung, Taiwan; ^2^Department of Occupational Therapy, College of Medical and Health Science, Asia University, Taichung, Taiwan; ^3^Department of Nursing, College of Pharmacy and Health Care, Tajen University, Pingtung, Taiwan; ^4^Department of Nursing, College of Health, National Taichung University of Science and Technology, Taichung, Taiwan; ^5^Department of Industrial and Information Management, National Cheng Kung University, Tainan, Taiwan

**Keywords:** sleep quality, internet dependence, Pittsburgh Sleep Quality Index, Internet Addiction Test, college students

## Abstract

**Background:**

Over 40% of Taiwanese College students experience sleep problems that not only impair their quality of life but also contribute to psychosomatic disorders. Of all the factors affecting the sleep quality, internet surfing is among one of the most prevalent. Female college students are more vulnerable to internet-associated sleep disorders than their male counterparts. Therefore, this study aims to investigate (1) the relationship between internet addiction and sleep quality, and (2) whether significant variations in sleep quality exist among students with different degrees of internet use.

**Methods:**

This structured questionnaire-based cross-sectional study enrolled students from a technical institute in southern Taiwan. The questionnaire collected information on the following three aspects: (1) demography, (2) sleep quality with Pittsburgh Sleep Quality Index (PSQI), and (3) severity of internet addiction using a 20-item Internet Addiction Test (IAT). Multiple regression analysis was performed to examine the correlation between PSQI and IAT scores among the participants. Logistic analysis was used to determine the significance of association between PSQI and IAT scores.

**Results:**

In total, 503 female students were recruited (mean age 17.05 ± 1.34). After controlling for age, body mass index, smoking and drinking habits, religion, and habitual use of smartphone before sleep, internet addiction was found to be significantly associated with subjective sleep quality, sleep latency, sleep duration, sleep disturbance, use of sleep medication, and daytime dysfunction. Worse quality of sleep as reflected by PSQI was noted in students with moderate and severe degrees of internet addiction compared to those with mild or no internet addiction. Logistic regression analysis of the association between scores on IAT and sleep quality, demonstrated significant correlations between quality of sleep and total IAT scores (odds ratio = 1.05:1.03 ∼ 1.06, *p* < 0.01).

**Conclusion:**

The results of this study demonstrated significant negative association between the degree of internet addiction and sleep quality, providing reference for educational institutes to minimize adverse effects associated with internet use and improve students’ sleep quality.

## Introduction

Adequate sleep is essential for growth hormone secretion that is required for normal physical development, particularly in adolescents. Previous studies revealed sleep problems in up to 40% of Taiwanese college students (Kang and Chen, 2009; Lin et al., 2018). With the increasing popularity of smartphones together with all its advanced technology, the use of the smartphone before sleep has become a habit for adolescents that could prolong sleep latency and decrease sleep duration (Yang et al., 2019). Of all the factors affecting sleep quality in college students, internet use is among one of the most prevalent. A previous study has shown that college students in Taiwan spend an average of up to 16.27 h per week on the Internet. Furthermore, the sleep quality of over half of those students was found to be adversely affected by the use of internet for chatting, playing games, and watching movies before sleeping (Lin et al., 2015). Previous studies have already underscored the link between internet use and sleep quality. A study investigating the correlation between internet use and sleep in 380 medical students concluded that overuse of mobile phones and social networks could impair sleep quality (Mohammadbeigi et al., 2016).

A number of studies have reported a poorer sleep quality in female college students than that in their male counterparts (Cheng et al., 2012; Surani et al., 2015; Saygın et al., 2016). Although the finding may be attributable to puberty-related female hormonal changes such as sleep quality impairment during menstruation (Baker and Driver, 2007; Orff et al., 2014), the increasing popularity of daily electronic product use (e.g., smartphones) also has an important part to play in sleep quality impairment in females (Hysing et al., 2015; Liu et al., 2018). The issue was further highlighted in another study showing a significantly higher mental impact of smartphone use on female adolescent students than that on males (Yang S.-Y. et al., 2018). The long-term indulgence in internet usage among college students could be attributed to the easy accessibility of their electronic devices to the internet that enables their participation in a variety of entertaining and social activities (e.g., online games, movies, and social media).

Regarding gender difference in susceptibility to internet-associated sleep disturbance, a study based on 4,750 adolescents has shown a significant increased risk in females compared to that in males (Yang J. et al., 2018). Together with the finding that female college students are more likely to develop internet dependence than male students (Chiu et al., 2013), the issue of the effect of internet addiction on sleep quality in female college students needs to be seriously addressed. The correlation between the extent of internet use and the quality of sleep among female college students needs more attention. In an attempt to elucidate the correlation between Internet addiction and sleep deprivation and to provide reference for minimizing inappropriate internet use to facilitate sleep quality improvement in female college students, the present study aimed at exploring (1) the correlation between internet addiction and sleep quality in female college students, and (2) whether variations in sleep quality exist among students with different degrees of internet use.

## Materials and Methods

### Study Design and Subject Recruitment

This cross-sectional study recruited college students from a technical institute in southern Taiwan who were required to complete a structural questionnaire between September 15 and November 15, 2018. Full explanation was given to all participants who were fully aware of their right to withdraw from participation anytime during the study period. Inclusion criteria were participants who were (1) female, (2) able to communicate in Mandarin Chinese, and (3) willing to complete the questionnaire. Individuals who were (1) pregnant or had children, (2) participating in night-time jobs at the time of this study, (3) unable to complete the questionnaire, or (4) diagnosed with psychiatric disorders regardless of severity were excluded from the present study. Ethical approval for the study was obtained from the National Cheng Kung University Human Research Ethics Committee (No. NCKU HREC-E-106-108-2). All participants gave their informed consents before participating in the present study. A total of 505 female college students were invited and returned written consents and were recruited into the study. Additionally, written informed consent was obtained from the parents or guardians of the participants who were under the age of 16. Sample size was estimated using G-power software (3.1.0). Based on a previous study in which R^2^ was reported to be 0.06 (Tan et al., 2016). 350 subjects were needed after estimating the condition of a type I error 0.05 to a power of 0.95.

### Research Instrument

All participants were required to complete a structural questionnaire that comprised of three parts. Because past studies have pointed out that some demographic variables such as smoking and drinking habits are predictors of sleep quality (Vargas et al., 2014; Warren et al., 2017; Hill et al., 2018; Kesintha et al., 2018; Yang et al., 2019), we collected variables that can affect sleep quality in Part One of the questionnaire. Part One included information on demography (i.e., age), anthropometry [i.e., body weight and height, body mass index (BMI)] as well as habits of tobacco consumption (defined as the smoking of at least one cigarette per day in the past 6 months) and alcohol consumption (defined as the drinking of alcoholic beverages at least once per week in the past 6 months), religious beliefs, and the habit of smartphone use before sleep (defined as smartphone use within 1 h before going to sleep at a frequency of at least five nights per week in the past 6 months).

Part Two of the questionnaire assessed the quality of sleep by using the Taiwanese version of the Pittsburgh Sleep Quality Index (PSQI) which is a parameter based on self-reported items in a questionnaire first developed by Buysse et al. (1989) and is one of the popular tools for the evaluation of sleep quality in the past 1 month. PSQI contained seven items each of which carries a score of 0 to 3 that signifies the frequency of each condition mentioned in each item, giving a range of scores between 0 and 21 (Buysse et al., 1989). The higher the score, the poorer the quality of sleep. The seven items assessed seven aspects of sleep quality: (1) Subjective sleep quality: Self-reported satisfaction with sleep quality in the past 1 month. The higher the score, the more unsatisfactory the subject felt; (2) Sleep latency: A higher score signifies a longer time required for falling asleep after going to bed; (3) Sleep duration: A higher score denotes a shorter sleep duration; (4) Habitual sleep efficiency: The higher the score, the lower the efficiency; (5) Sleep disturbances: The higher the score, the more severe the disturbance; (6) Use of sleep medication: A higher score represents a more frequent requirement; and (7) Daytime dysfunction: The higher the score, the more problems one encounters when engaging in daily activities (e.g., staying awake while driving, eating, or participating in social activities (Buysse et al., 1989). In the current study, the PSQI score >5 or PSQI score ≤5 was chosen to distinguish the quality of sleep in each student. A total score of ≤5 represents satisfactory quality of sleep, whilst that of >5 signifies a poor quality of sleep (Buysse et al., 1989). The Taiwanese version of PSQI has been shown to have good reliability and validity (Tsai et al., 2005). The Cronbach’s α coefficients of the PSQI were 0.74 in this study.

Part Three evaluated the severity of internet addiction through the adoption of the 20-item Internet Addiction Test (IAT) first proposed by Young (1998). Because of the popularity of IAT in assessing the degree of internet use among adults and adolescents, it has been translated into different languages (Widyanto and McMurran, 2004), and the Taiwanese version of IAT has been widely used in adolescents with good psychometrics (Dhir et al., 2015). Each of the 20 items in IAT carries a score on a Likert scale of 0 (i.e., not applicable) to 5 (i.e., always), giving a total score ranging from 0 to 100. The higher of the score, the more severe the internet addiction. There are four degrees of internet addiction according to the IAT score: A score of 0–30 reflects normal internet use, while a score of 31–49 denotes mild degree of internet addiction. At the other end of the spectrum, a score of 50–79 indicates moderate level of internet addiction, whilst a score of 80–100 signifies severe dependence upon the Internet (Young, 1998). IAT assessed six patterns of symptoms, including (1) Salience (5 items): The higher the score, the more likely that the participant focuses on the internet with possible indifference to other activities and/or relationships. A sense of boredom and emptiness may arise if internet is not available; (2) Excessive Use (5 items): The score is positively associated with excessive internet use which is defined as staying on the internet for a duration longer than expected. The individual may feel panic or frustration after prolonged inaccessibility to the internet; (3) Neglect Work (3 items): A higher score reflects the probability that the participant tends to consider the internet to be something indispensable in daily life comparable to television or telephone on which spending a considerable amount of time is justified, resulting in impaired academic or work performance as well as overall productivity; (4) Anticipation (2 items): The higher the score, the stronger the anticipation of internet access; (5) Lack of Control (3 items): A higher score indicates probable lack of self-control over the amount of time spent on the internet and that may cause annoyance to others; and (6) Neglect Social Life (2 items): A higher score suggests that the participants are likely to neglect their real-world social life and prefer to participate in social activities on the internet. Psychometric analysis has shown satisfactory reliability and validity of IAT (Widyanto and McMurran, 2004). The Cronbach’s α coefficients of the IAT were 0.93 in this study.

### Statistical Analysis

The statistical software of SPSS version 22.0 for Mac was used for the whole study. The participants were divided into two groups: those with PSQI score ≤5 (i.e., satisfactory sleep quality) and those with PSQI score >5 (i.e., poor sleep quality) according to descriptive statistics of demographic variables. Student’s *t*-test (continuous variables) and Fisher’s Exact Test (categorical variables) were used to analyze the significance of difference in demographic data and IAT scores between the two groups, while one-way ANOVA was utilized for determining the significance of difference in total PSQI scores and sub-scores on the seven aspects of sleep quality among normal internet users (0–30) as well as those with mild (31–49) and moderate to severe (50–100) internet addiction. *Post hoc* was tested using Tukey adjustment. After setting total PSQI scores and sub scores on the seven items as dependent variables as well as total IAT scores and scores on the six patterns of symptoms as independent variables, multiple regression analysis was performed to examine the correlation between PSQI and IAT scores among the participants. Because age (Hsieh et al., 2018; Kesintha et al., 2018), BMI (Canan et al., 2014; Vargas et al., 2014), smoking and drinking habits (Lee and Lee, 2017; Warren et al., 2017), religion (Hill et al., 2018; Nadeem et al., 2018), habitual use of smartphone before sleep (Ayar et al., 2017; Wang et al., 2019) might have impacted on sleep quality and internet addiction, thus these variables were controlled during analysis. Finally, logistic analysis was used to determine the significance of association between PSQI and IAT scores after setting the quality of sleep (i.e., 0: good; 1: poor) as dependent variable, while setting total IAT scores and scores on the six patterns of symptoms as independent variables after controlling for confounding factors. Average values are expressed as mean ± standard deviation (SD). A *p* value of less than 0.05 is considered statistically significant. In addition, before all regression analysis, preliminary analyses were conducted to ensure that no violation of the assumptions of normality, linearity and multicollinearity existed.

## Results

### Demographic, Anthropometric, and Lifestyle Characteristics as Well as Sleep Quality and Level of Internet Addiction

In total, 503 female college students who completed the questionnaire were recruited for the current study, and two participants were excluded due to questionnaires incompleteness. The demographic, anthropometric, and lifestyle characteristics of the participants as well as their scores on internet addiction are shown in Table 1. The mean age of the participants was 17.30 ± 1.34 (range, 15 – 22). Over 95% had no habit of tobacco or alcohol consumption. On the other hand, over 95% of participants reported habitual smartphone use before bedtime. The prevalence of a smoking habit was significantly higher in students with poor sleep quality than those with good quality of sleep (*p* < 0.01) (Table 1). According to IAT scores, normal Internet users and those with mild degrees of Internet dependence comprised over 80% of the study population. Students with good quality of sleep had significantly lower IAT score totals compared to the scores of those with bad sleep quality (*p* < 0.01). Consistently, with the exception of neglect of work, scores on the other five patterns of symptoms were significantly lower in participants with good sleep quality than the respective scores in those with poor sleep quality (all *p* < 0.01).

**Table 1:** Demographic, anthropometric, and lifestyle characteristics as well as sleep quality and level of internet addiction.

		Sleep quality		*p*
	Total *N* = 503	Good (*n* = 229)	Poor (*n* = 274)
Age (mean ± SD)	17.30 ± 1.34	17.31 ± 1.31	17.29 ± 1.36	0.83^b^
BMI (mean ± SD)	20.57 ± 4.08	20.30 ± 3.45	20.79 ± 4.54	0.18^b^
**Smoking habit (n, %)**				0.01^a,∗^
No	482 (95.80%)	225 (98.30%)	257 (93.80%)	
Yes	21 (4.20%)	4 (1.70%)	17 (6.20%)	
**Drinking habit (n, %)**				0.05^a^
No	485 (96.40%)	225 (98.30%)	260 (94.90%)	
Yes	18 (3.60%)	4 (1.70%)	14 (5.10%)	
**Religion (n, %)**				0.42^a^
No	258 (51.30%)	122 (53.30%)	136 (49.60%)	
Yes	245 (48.70%)	107 (46.70%)	138 (50.40%)	
**Smartphone use before sleep (n, %)**				0.67^a^
No	22 (4.40%)	11 (4.80%)	11 (4.00%)	
Yes	481 (95.60%)	218 (95.20%)	263 (96.00%)	
**IAT level (n, %)**				
Normal	164 (32.60%)	102 (44.50%)	62 (22.60%)	<0.01^a∗^
Mild	265 (52.70%)	103 (45.00%)	162 (59.10%)	
Moderate	70 (13.90%)	22 (9.60%)	48 (17.50%)	
Severe	4 (0.80%)	2 (0.90%)	2 (0.70%)	
**Symptoms (mean ± SD)**				
Total score	37.34 ± 12.00	34.34 ± 11.32	39.85 ± 11.99	<0.01^b∗^
Salience	7.58 ± 3.10	6.70 ± 2.87	8.32 ± 3.10	<0.01^b∗^
Excessive use	10.22 ± 3.36	9.34 ± 3.09	10.96 ± 3.41	<0.01^b∗^
Neglect of work	4.78 ± 1.86	4.61 ± 1.86	4.92 ± 1.84	0.06
Anticipation	3.64 ± 1.51	3.42 ± 1.38	3.82 ± 1.58	<0.01^b∗^
Lack of control	5.66 ± 2.18	5.16 ± 2.05	6.08 ± 2.20	<0.01^b∗^
Neglect of social life	4.20 ± 1.71	3.88 ± 1.61	4.47 ± 1.75	<0.01^b∗^

*^∗^p < 0.05; ^a^Significance of difference determined using Fisher’s exact test; ^b^Significance of difference determined using Student *t*-test; BMI, Body mass index; and IAT, Internet Addiction Test.*

### Sleep Quality in Participants With Different Degrees of Internet Addiction

ANOVA on PSQI total scores and scores on the different components in students with different degrees of internet addiction revealed significant lower scores in normal internet users and those with mild internet dependence compared to those with moderate to severe internet addiction on all items with the exception of habitual sleep efficiency (Table 2). The results of significant differences between the two groups in *post hoc* were also shown in Table 2. In the total scores, subjective sleep quality and sleep latency of PSQI, the mean scores in moderate to severe internet addiction users were significantly higher than those in mild internet dependence users and normal internet users; the mean scores in mild internet users were significantly higher than those in normal internet users (Table 2).

**Table 2:** The Pittsburgh Sleep Quality Index (PSQI) total scores and scores on its different components in students with different degrees of internet addiction.

	Normal (a)		Mild (b)		Moderate to severe (c)		*p*	*Post hoc*
Components	Mean	SD	Mean	SD	Mean	SD		Tukey
Total score	4.97	2.90	6.49	2.94	6.85	2.91	<0.01^∗^	(c) > (b) > (a)
Subjective sleep quality	0.94	0.80	1.24	0.78	1.28	0.77	<0.01^∗^	(c) > (b) > (a)
Sleep latency	0.72	0.78	1.15	0.86	1.00	0.89	<0.01^∗^	(c) > (b) > (a)
Sleep duration	0.81	0.95	0.97	0.96	1.14	1.04	0.04^∗^	(c) > (a)
Habitual sleep efficiency	0.18	0.50	0.23	0.63	0.26	0.62	0.56	–
Sleep disturbance	0.93	0.58	1.19	0.57	1.14	0.58	<0.01^∗^	(b) > (a); (c) > (a)
Use of sleep medication	0.06	0.35	0.08	0.42	0.22	0.56	0.02^∗^	(c) > (b); (c) > (a)
Daytime dysfunction	1.34	0.92	1.62	0.85	1.82	0.80	<0.01^∗^	(b) > (a); (c) > (a)

*^∗^p < 0.05; ^∗∗^p < 0.01.*

### Association Between Internet Addiction and Sleep Quality

The VIFs were between 1.02∼1.03 and therefore considerably lower than the recommended threshold of 10, suggesting that multicollinearity did not exist. In addition, the study also used linearity regression method to test the relationships of the two variables with raw data. The result of Table 3 was supported by Appendixes 1, 2, which present that each symptom in Internet addiction is significantly associated with sleep quality. From an overall point of view, the results of linearity regression analysis in Appendix 1 can reveal important data for this study.

**Table 3:** Regression coefficients from multiple regression analysis on associations of the six patterns of symptoms in Internet Addiction Test (IAT) with the seven items on sleep quality in Pittsburgh Sleep Quality Index (PSQI)^†^.

PSQI IAT	Total score	Subjective sleep quality	Sleep latency	Sleep duration	Habitual sleep efficiency	Sleep disturbance	Use of sleep medication	Daytime dysfunction
Total score	0.06^∗∗^	0.01^∗∗^	0.01^∗∗^	0.01^∗^	<0.01	0.01^∗∗^	0.01^∗∗^	0.02^∗∗^
Salience	0.30^∗∗^	0.05^∗∗^	0.05^∗∗^	0.05^∗∗^	0.02	0.04^∗∗^	0.02^∗∗^	0.07^∗∗^
Excessive use	0.20^∗∗^	0.03^∗∗^	0.03^∗∗^	0.03^∗∗^	0.01	0.02^∗∗^	0.02^∗∗^	0.06^∗∗^
Neglect of work	0.14	0.02	0.01	0.01	0.01	0.02	0.05^∗∗^	0.03
Anticipation	0.27^∗∗^	0.03	0.06^∗^	0.02	0.01	0.05^∗∗^	0.03^∗^	0.07^∗∗^
Lack of control	0.31^∗∗^	0.05^∗∗^	0.05^∗∗^	0.05^∗^	0.02	0.05^∗∗^	0.02	0.08^∗∗^
Neglect of social life	0.25^∗∗^	0.05^∗^	0.04	0.05	<0.01	0.03	0.02	0.06^∗∗^

*^†^Controlled for age, body mass index (BMI), smoking and drinking habits, religion, habitual use of smartphone before sleep; ^∗^p < 0.05; ^∗∗^p < 0.01.*

The results of multiple regression analysis demonstrated significant associations of IAT total scores with the total score of PSQI and the sub-scores on its six items, except the item of habitual sleep efficiency (Table 3). Likewise, the total score of PSQI was significantly correlated with the scores on five of the six symptom patterns of internet addiction (all *p* < 0.01) with the exception of neglect of work. Focusing on the associations between different components of sleep quality and the symptom patterns of internet addiction, sleep latency was significantly related to four symptom patterns including salience (*p* < 0.01), excessive use (*p* < 0.01), anticipation (*p* < 0.05), and lack of control (*p* < 0.01). On the other hand, sleep duration was associated with three symptom patterns of internet addiction, namely, salience (*p* < 0.01), excessive use (*p* < 0.01), and lack of control (*p* < 0.05). Interestingly, habitual sleep efficiency was the only item on PSQI that showed no significant correlation with any symptom patterns of internet addiction (Table 3). With the exceptions of neglect of work and neglect of social life, there were significant correlations between sleep disturbance and the other four symptom patterns. In addition, use of sleep medication among the participants was associated with four of the symptom patterns of internet addiction except lack of control and neglect of social life, while daytime dysfunction was significantly related to all symptom patterns of IAT except neglect of work.

Table 4 shows that the odds ratio was 1.05, and the 95% confidence interval was 1.03∼1.06 as a risk ratio. It means that Internet addiction influenced the poor sleep quality group (coded 1) more seriously than the good sleep quality group (coded 0). After controlling for confounding variables including age, BMI, smoking and drinking habits, religion, and habitual use of smartphone before sleep (Table 4), logistic regression analysis of the association between scores on IAT and sleep quality demonstrated significant correlations between quality of sleep and total score of IAT (B = 0.04, S.E.: 0.01, *p* < 0.01) as well as sub-scores on five of the symptom patterns, namely, salience (B = 0.20, S.E.: 0.04, *p* < 0.01), excessive use (B = 0.16, S.E.: 0.03, *p* < 0.01), anticipation (B = 0.19, S.E.: 0.06, *p* < 0.01), lack of control (B = 0.22, S.E.: 0.05, *p* < 0.01), and neglect of social life (B = 0.22, S.E.: 0.06, *p* < 0.01). The results indicated that the degree of Internet addiction (including five patterns of symptoms) was positively associated with poor sleep quality. In other words, the participants with poor sleep quality were significantly more likely to show these symptom patterns of Internet addiction than those with good sleep quality.

**Table 4:** Logistic regression analysis on association between scores on Internet Addiction Test (IAT) and sleep quality^†^.

Item	B	S.E.	OR (95% CI)	*p*
Total score	0.04	0.01	1.05 (1.03 – 1.06)	<0.01^∗^
Salience	0.20	0.04	1.22 (1.14 – 1.30)	<0.01^∗^
Excessive use	0.16	0.03	1.17 (1.10 – 1.25)	<0.01^∗^
Neglect of work	0.09	0.05	1.10 (0.99 – 1.21)	0.07
Anticipation	0.19	0.06	1.21 (1.07 – 1.38)	<0.01^∗^
Lack of control	0.22	0.05	1.24 (1.13 – 1.36)	<0.01^∗^
Neglect of social life	0.22	0.06	1.24 (1.11 – 1.39)	<0.01^∗^

*^†^Controlled for age, body mass index (BMI), smoking and drinking habits, religion, and habitual use of smartphone before sleep; ^∗^p < 0.05; B, regression coefficient; S.E., standard error; OR, odds ratio; and CI, confidence interval.*

## Discussion

The results of the present study showed unsatisfactory quality of sleep in over half of the participants (54.5%) and that those with a smoking habit and a higher level of Internet addiction tended to have poorer sleep quality. In addition, comparison of the scores of Internet addiction between participants with good quality of sleep and those with poor sleep quality revealed significantly lower scores on five out of six of the symptom patterns in the latter, indicating that those with poor sleep quality tended to focus on internet activities, use the internet excessively, have strong anticipation of internet access, exhibit a lack of self-control over the amount of time spent on the internet, and participated in social activities on the internet while neglecting real-world social life. The result was similar with a previous study showing a negative impact of excessive internet use on sleep quality (Mohammadbeigi et al., 2016). Furthermore, the present study also identified the correlations between quality of sleep and five symptom patterns of internet addiction, namely, salience, excessive use, anticipation, lack of control, and neglect of social life. Previous studies have shown that not only does internet addiction adversely affect the quality of sleep in college students (Lin et al., 2018) but it also impairs sleep quality in children (Chen and Gau, 2016), adolescents as a whole (Chen and Gau, 2016), and adults (Bakken et al., 2009). Internet addiction has been found to contribute to disturbed circadian rhythm (Chen and Gau, 2016) that may negatively influence bedtime and sleep duration, leading to daytime fatigue and impaired work performance.

Additionally, the current study showed that 14% of the recruited female college students were at moderate to severe levels of Internet addiction. The above phenomenon was similar to a report in a previous study which was conducted on 3,616 Taiwanese college students (Lin et al., 2011). Factors contributing to the popularity of participation in social activities on the Internet among college students including the anonymous nature of the social media without requirement for showing one’s physical appearance, no limitations of time and space, and low risk offers an ideal way of alleviating loneliness and establishing social networks, thereby elevating the risks of excessive Internet use and Internet addiction (McKenna and Bargh, 2000). Besides, college students tend to entertain themselves through online games and/or shopping as well as chatting on social media which provides pleasure and refuge from daily pressure, mental stress, anxiety, or loneliness (Morahan-Martin and Schumacher, 2003). While poor sleep quality is closely associated with daily life behaviors including Internet over usage (Mohammadbeigi et al., 2016). In terms of overuse, internet behaviors (such as online gaming) could lead to sleep problems such as sleep deprivation (An et al., 2014).

Regarding the correlation between sleep quality and IAT, our results demonstrated significant associations of total IAT score with the total score of PSQI as well as the sub scores on its six items (except habitual sleep efficiency) (Table 3). This finding is supported by that of a previous study on the correlation between mood status and sleep quality (Chang et al., 2017). The results of the present study also implicated that an excessive use of Internet could be linked to impaired sleep quality, prolonged sleep latency, shortened sleep duration, sleep disturbances, need for sleep medication, and daytime dysfunction, further highlighting the adverse impact of Internet addiction on different parameters regarding sleep quality. Consistently, logistic regression analysis of the current study demonstrated significant associations between sleep quality and most of the IAT items (Table 4). On the other hand, a previous study has shown that subjective perception of sleep quality is related to the individual’s sleep attitude (Peach et al., 2018). This may help interpret our findings that a poor sleep attitude as reflected by excessive use of the Internet, lack of control, anticipation of Internet access, and indulgence in online social activities would have a negative impact on an individual’s subjective perception of sleep quality.

One of the interesting findings of the present study was the prolonged sleep latency in subjects with moderate to severe Internet addiction. The result was not surprising after taking into account the high prevalence of Internet access through smartphones or tablet computers among Taiwanese college students (Yang et al., 2019) and the stimulation of the central nervous system through participating in games and watching action movies online before sleep which are known contributors to prolonged sleep latency (Higuchi et al., 2005). Another possible explanation is the emission of blue light through the screens that is known to suppress melatonin secretion from the pineal gland, leading to prolongation of sleep latency (Moderie et al., 2017).

Another accompanying finding in the current study was the shortened sleep duration among those with moderate to severe Internet addiction (Table 3). A large-scale study investigating the link between Internet use and self-reported sleep duration in up to 130,000 South Korean adolescents between the ages of 13 and 18 has identified a significant negative association between Internet use and sleep duration (Do et al., 2013). Another study on the risk factors associated with short sleep duration that involved twenty thousand Chinese children ranging in ages from five to eleven also reached the conclusion that Internet use is a significant predictor of shortened sleep duration (Li et al., 2010). The negative psychological and physiological impacts of inadequate sleep have been well-documented, including negative emotions (Gujar et al., 2011), anxiety (Alfano et al., 2006), obesity (Wang et al., 2018), and increased risks of participating in hazardous behaviors (e.g., tobacco and/or alcohol consumption, driving while under the influence) (Weaver et al., 2018). On the other hand, a significant association between sleep duration and health responsibility has also been reported (Yang et al., 2019). Excessive Internet users and those with poor self-control over internet use, who were found to have shortened sleep durations in the current study, could have poor health responsibility that may be reinforced through education.

The item of sleep disturbance on PSQI is a measure of interferences during sleep such as the need for going to the toilet, troubled breathing, coughing, feeling hot or cold, having nightmares or pain. A high score on this item may imply a physical impairment or discomfort (Schlarb et al., 2017). The high scorers on the items of salience, excessive use, neglect of work, anticipation, or lack of control in the present study also tended to relate to sleep disturbance from physical factors. Therefore, the finding may suggest a negative impact from Internet use on physical health. As previous investigation has shed light on, the fact that the negative influence of excessive internet use on normal physiological functions of the body including the manifestations of pain and physical discomfort may impair academic and/or work performance (Kelley and Gruber, 2012).

Use of sleep medications is one of the most common and effective ways to tackle sleep problems (Homsey and O’connell, 2012). The identification of a positive correlation between the level of Internet addiction and use of sleep medication in the present study may imply that those with moderate and severe Internet addiction tend to take sleep medication. The finding may be explained by the highly significant link between internet addiction and negative emotions (e.g., depression, anxiety, interpersonal sensitivity, and hostility) (Chou et al., 2017; Kim et al., 2018) as well as the positive association between emotional disturbance and the use of sleep medications (Kodaira and Silva, 2017).

Furthermore, the results of the current study demonstrated a link between Internet use and daytime dysfunction, suggesting a negative impact of Internet overuse on daily activities such as driving (cycling), eating regular meals, or participating in social activities during daytime hours. The significant association of daytime dysfunction with the items of anticipation and neglect of social life in this study, may also imply daytime functional impairment from empty anticipation and neglect of a real-world social life due to inaccessibility to the Internet in daytime when an individual has to participate in normal academic or work activities. As a whole, previous studies have shown that normal Internet use (Mohammadbeigi et al., 2016), avoidance of emotional fluctuations before sleep such as participating in online activities (Higuchi et al., 2005), and maintenance of good bedtime hygiene such as abstinence from excessive tobacco or alcohol use as well as sticking to a regular bedtime (Warren et al., 2017) are all positive contributors to sleep quality.

The logistic regression demonstrated the correlations between sleep quality and internet addiction (Table 4). Further review of the subscale of IAT, reveals that both “lack of control” and “neglect of social life” have a greater impact on sleep quality, while “neglect of work” has the least impact on sleep quality. This implies that internet users who lack self-control may suffer a lack of sleep quality due to poor-control behavior of Internet usage (such as delaying bedtime); users who neglect of social life in real-life may lack positive social support which can lead to poor sleep quality (Stafford et al., 2017). A previous study (Bonnefond et al., 2006) also reported that individuals with a low social life such as night-shift workers had poor sleep quality. Definitely, further research is required.

### Limitations

The present study had several limitations that have to be taken into account for accurate interpretation of its findings. First, although self-reported sleep parameters such as duration are frequently used (Campbell et al., 2013), the lack of objective evidence to support data accuracy introduced potential bias into the study. Second, the recruitment of students from a single educational institute, which is a medicine and management college, may limit the extrapolation of the findings. Additionally, the present study excluded participants who were pregnant or had children, while future researchers could choose pregnant women and those women who have children as subjects. Third, the choice of the period of study for the current research during the semester could not reflect the participants’ status of Internet use and sleep patterns during long holidays. Fourth, this study did not collect the diagnosis of sleep quality among participants, so there is no cut-off point of sleep quality based on clinical diagnosis, which may also limit the generalization of the results in this study. Finally, the cross-sectional design of the current study precluded the establishment of any cause-and-effects relationships among the study parameters. Nevertheless, the present study, which investigated the association between Internet addiction and sleep quality in female college students through analyzing the correlations among the parameters on sleep quality and the symptom patterns of Internet addiction, may provide useful information for educational institutes and related professionals to design suitable programs that can deal with Internet addiction for enhancing sleep quality and academic performance among female college students. Moreover, further research is recommended to recruit students of different genders to explore gender differences in detail.

## Conclusion

The results of this study demonstrated significant associations of Internet addiction with most parameters of sleep quality (i.e., subjective sleep quality, sleep latency, sleep duration, sleep disturbance, use of sleep medication, and daytime dysfunction), highlighting a poorer quality of sleep in those with moderate and severe Internet addiction than that in normal internet users and those with mild internet dependency. Moreover, Internet addiction was also found as a significant predictor of poor sleep quality. Consequently, preoccupation with the Internet, Internet overuse, anticipation of Internet access, loss of self-control over the time spent on the Internet, and indulgence in online social activities are strongly not recommended among female college students due to all the negative contributors to sleep quality.

## Data Availability

All datasets generated for this study are included in the manuscript and/or the Supplementary Files.

## Ethics Statement

Ethical approval for the study was obtained from the National Cheng Kung University Human Research Ethics Committee (No. NCKU HREC-E-106-108-2).

## Author Contributions

P-HL and S-YY designed the survey used in this study, led coding, analysis, and wrote the first draft. Y-CL, K-LC, Y-LL, and P-LH were responsible for the conceptualization of the study, assisted in data analysis, and reviewed this manuscript for contextual content. S-YY and P-LH critically reviewed the manuscript. P-HL and Y-CL assisted in data collection.

## Conflict of Interest Statement

The authors declare that the research was conducted in the absence of any commercial or financial relationships that could be construed as a potential conflict of interest.
