# Supplementary material for: The Relationship Between Sleep Quality and Internet Addiction Among Female College Students
Source: Front Neurosci. 2019 Jun 12;13:599. doi: 10.3389/fnins.2019.00599 (PMC6582255; doi:10.3389/fnins.2019.00599)
Supplement: Supplementary file 1 [file Data_Sheet_1.docx]

**Appendix 1**. Results of linearity regression analysis, multicollinearity condition index, and VIF with raw data

|  | Dependent variable: Sleep quality | | | Multicollinearity condition index | VIF |
| --- | --- | --- | --- | --- | --- |
|  | Path coefficient | T value | P value |  |  |
| Salience | 0.21 | 5.58 | < 0.01 | 4.23 | 1.01 |
| Excessive use | 0.23 | 5.22 | < 0.01 | 4.23 | 1.02 |
| Neglect work | 0.30 | 6.97 | < 0.01 | 3.59 | 1.03 |
| Anticipation | 0.50 | 13.01 | < 0.01 | 2.10 | 1.03 |
| Lack of control | 0.18 | 4.21 | < 0.01 | 3.83 | 1.03 |
| Neglect social life | 0.25 | 5.83 | < 0.01 | 3.24 | 1.02 |

Note: All control variables are is insignificant, such as age, body mass index (BMI), religion, smoking, drinking habits, and habitual use of smartphone before sleep. All multicollinearity condition indexes are smaller than 30 and all VIFs are smaller than 10, indicating the absence of serious collinearity problems among the independent variables.

**Appendix 2**. Results of the relationship between Internet addiction and sleep quality (raw data)

Internet addiction

Salience

Excessive use

Neglect work

Anticipation

Lack of control

Control variables:

Age, body mass index (BMI), religion, smoking, drinking habits, and habitual use of smartphone before sleep

Neglect social life

Sleep quality

0.214***

0.227***

0.299***

0.503***

0.252***

0.184***

Note: ***p <0.001
